# Structure-Based Virtual Screening and Biological Evaluation of Peptide Inhibitors for Polo-Box Domain

**DOI:** 10.3390/molecules25010107

**Published:** 2019-12-27

**Authors:** Fang Yan, Guangmei Liu, Tingting Chen, Xiaochen Fu, Miao-Miao Niu

**Affiliations:** Department of Pharmaceutical Analysis, China Pharmaceutical University, Nanjing 210009, China; cpuyanfang@hotmail.com (F.Y.); 2020141241@stu.cpu.edu.cn (G.L.); jyjq23@163.com (T.C.); demded17q@126.com (X.F.)

**Keywords:** virtual screening, polo-like kinase, polo-box domain, peptide inhibitor, cancer therapy

## Abstract

The polo-box domain of polo-like kinase 1 (PLK1-PBD) is proved to have crucial roles in cell proliferation. Designing PLK1-PBD inhibitors is challenging due to their poor cellular penetration. In this study, we applied a virtual screening workflow based on a combination of structure-based pharmacophore modeling with molecular docking screening techniques, so as to discover potent PLK1-PBD peptide inhibitors. The resulting 9 virtual screening peptides showed affinities for PLK1-PBD in a competitive binding assay. In particular, peptide 5 exhibited an approximately 100-fold increase in inhibitory activity (IC_50_ = 70 nM), as compared with the control poloboxtide. Moreover, cell cycle experiments indicated that peptide 5 effectively inhibited the expression of p-Cdc25C and cell cycle regulatory proteins by affecting the function of PLK1-PBD, thereby inducing mitotic arrest at the G2/M phase. Overall, peptide 5 can serve as a potent lead for further investigation as PLK1-PBD inhibitors.

## 1. Introduction

Cancer is a highly fatal disease characterized by uncontrolled cell proliferation, which has led to the death of millions of people. Specifically, 9.6 million people died from cancer in 2018 and 18.1 million cases were newly diagnosed [1]. The centrosome is an important organelle in the cell cycle process, which is responsible for the separation of genetic material, and its deregulation is related to the appearance of cancer [2]. Polo-like kinases (PLKs) are a subfamily of serine–threonine protein kinases that consist of multiple isoforms (PLK1 to PLK4), and play crucial roles in centrosome amplification [3,4,5]. Among the PLKs, PLK1 is an essential regulator for the maturation of the centrosome and previous studies have shown that it is overexpressed in a wide range of cancers, which makes it an attractive target for anticancer drug design [5,6,7].

The structure of PLK1 consists of an N-terminal kinase catalytic domain (KD) and a C-terminal polo-box domain (PBD) [8,9]. Targeting PLK1-PBD helps to avoid the selectivity issue of ATP competitive inhibitors resulting from the conservative structure of the kinase binding site, because the PBD is featured only for the PLK1 family [9]. Nevertheless, it is difficult for small molecules to regulate this process, owing to the large interfaces between proteins [10]. In contrast to conventional small molecules, therapeutic peptides are relatively safe and well-tolerated [11,12]. Thus, some phosphopeptide inhibitors of PLK1-PBD have been synthesized based on the fact that the PBD can specifically bind to the serine- or threonine-phosphorylated peptides (p-S/T motif) of Cdc25C [13,14]. However, due to the di-anionic motif, the clinical use of the PLK1-PBD inhibitors has been limited by their poor cellular penetration. Therefore, there is a pressing need to develop effective PLK1-PBD inhibitors.

Recent studies have shown that pharmacophore-based drug design has become an important tool in drug discovery [15,16]. In this work, we constructed a pharmacophore model based on three X-ray crystallographic structures of the PLK1-PBD and further validated these using the Gunner–Henry (GH) score method. The generated pharmacophore model was used as a query to screen potent PLK1-PBD peptide inhibitors from the peptide database. Subsequently, the retrieved peptides were filtered by molecular docking studies. Finally, nine peptides were identified as potential leads based on the calculated binding free energies and favorable binding interactions. 

## 2. Results and Discussion

### 2.1. Pharmacophore Modeling

**Figure 1:**
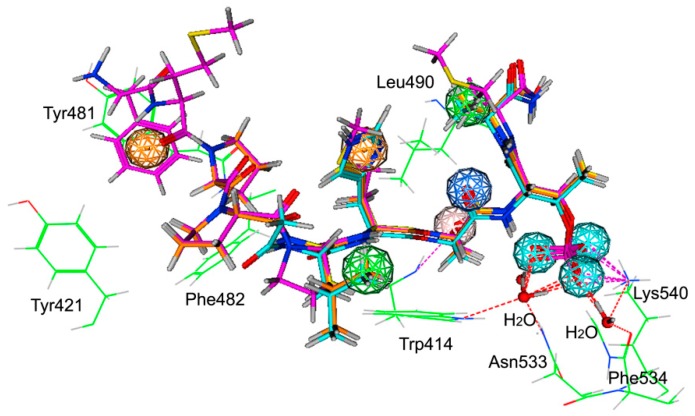
The generated pharmacophore model in the binding site of PLK1 polo-box domain (PBD). The bound ligands are included in the pharmacophore modeling. Pharmacophore features are color-coded: Green, two hydrophobic feature (F1 and F8: Hyd); cyan, three anionic and hydrogen bond acceptor features (F2–F4: Ani&Acc); blue, one hydrogen bond acceptor feature (F5: Acc); pink, one hydrogen bond donor and acceptor feature (F7: Don&Acc); orange, two aromatic features (F6 and F9: Aro). Active site residues (green) are shown in line form; red balls mean water molecules.

In this study, we have created a structure-based pharmacophore model of PLK1-PBD using different starting structures. We have chosen three X-ray crystallographic structures of the PLK1-PBD available in the Protein Data Bank (PDB) database (accession codes: 3Q1I, 3P36, and 3FVH). These structures were crystallized independently and have a high resolution of less than 2 Å. The generated structure-based pharmacophore model included nine features (Figure 1): Two hydrophobic features (F1 and F8: Hyd), three anionic and hydrogen bond acceptor features (F2–F4: Ani&Acc), one hydrogen bond acceptor feature (F5: Acc), one hydrogen bond donor and acceptor feature (F7: Don&Acc), and two aromatic features (F6 and F9: Aro). The features of the model were found to directly correspond to some key amino acids including Asn533, Phe534 and Lys540, Trp414, Leu490, Tyr481, Tyr421 and Phe482, which play a critical role in PLK1-PBD inhibition activity [17,18]. As shown in Figure 1, the phosphate groups of ligands that formed multiple direct and water-mediated hydrogen-bond interactions with Asn533, Phe534 and Lys540 mapped the F2–F4 features. The hydroxyl groups of the ligands showing a hydrogen-bond interaction with Trp414 overlaid the F7 feature, while the hydrophobic groups that enabled considerable interactions with Leu490 and Trp414 mapped the F1, F6 and F8 features, respectively. Moreover, the benzene ring of the ligand that forms a very important hydrophobic interaction with a hydrophobic pocket including Tyr481, Tyr421 and Phe482 mapped the F9 feature, and their oxygen atom mapped the F5 feature. The mapping of bound ligands on the pharmacophore model is shown in Figure 1.

### 2.2. Validation and Database Screening

The quality of the generated structure-based model was assessed using the GH score as a metric to search a database that consisted of 1985 inactive and 15 active molecules. A set of statistical parameters such as total hits (*Ht*), % ratio of actives, % yield of actives, enrichment factor (*E*), false negative, false positives, and goodness-of-hit score (*GH*) were calculated (Table 1). The *E* value for pharmacophore model was 96, as it identified 13 active hits from 18 molecules. The higher the *E* value, the greater the ability of a pharmacophore in identifying the active molecules. These validation results demonstrate that the pharmacophore model is very efficient for database screening. When a GH score is higher than 0.7, the model is very good. It was observed to be 0.76 for the pharmacophore model, which indicates a good ability to distinguish the active from the inactive molecules.

**Figure 2:**
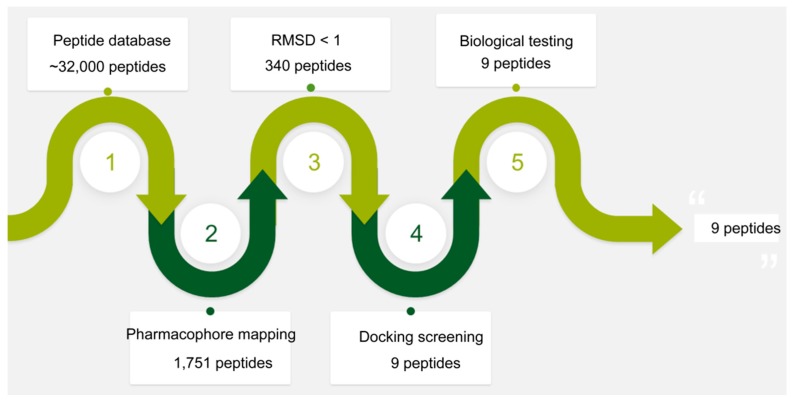
A workflow overview of pharmacophore modeling, selection of compounds, and biological testing.

The flowchart of virtual screening used in this study is shown in Figure 2. To confirm the discriminatory ability of the generated pharmacophore model, the pharmacophore model was firstly used as a 3D query to identify potential peptide inhibitors from the database, containing ~32,000 peptides. According to the root mean square distance (RMSD) value less than 1 Å, 340 selected peptides were further docked into the PLK1-PBD active site. The docking scores between PLK1-PBD and 340 peptides were calculated by the dG docking scoring function of the molecular operating environment (MOE) (lower docking scores indicate better binding affinity). Considering a cutoff to classify compounds as active and inactive, we used a −20 kcal/mol cutoff in docking score to prune the hit list. Among 340 peptides, 9 peptides (peptides 1–9) with docking scores less than −20 kcal/mol were finally selected for biological testing (Table 2). Figure S1 depicts a good pharmacophore mapping of 9 peptides on Hypo1.

### 2.3. PLK1-PBD Inhibition Assay

To test the binding ability of 9 peptides to the PLK1-PBD, a competitive fluorescence polarization (FP) assay was performed (Table S1). These selected peptides exhibited stronger inhibition activities (IC_50_ < 1 μM) towards PLK1-PBD than the control poloboxtide. In particular, peptide 5, as the most potent inhibitor (IC_50_ = 0.07 μM), showed an approximately 100-fold increase in inhibitory activity. The results indicated that the integrated virtual screening procedure had a great potential for identification of PLK1-PBD inhibitors. It was highly selective for PLK1-PBD. As shown in Table S2, the peptide 5 exhibited minimal inhibition of PLK2-PBD and PLK3-PBD tested (≤10% inhibition of PLK2-PBD or PLK3-PBD at 1 μM inhibitor concentration).

**Figure 3:**
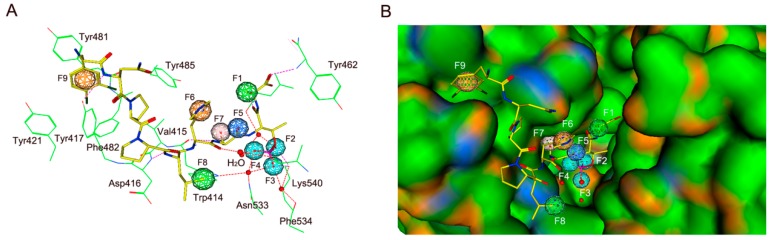
(**A**) The 3D ligand–protein interaction diagram and pharmacophore mapping of peptide 5 in the binding site of PLK1-PBD (PDB ID: 3Q1I). (**B**) Key interactions involved in stabilizing peptide 5 in the binding site. Pharmacophore features are color-coded: Green, two hydrophobic features (F1 and F8: Hyd); cyan, three anionic and hydrogen bond acceptor features (F2–F4: Ani&Acc); blue, one hydrogen bond acceptor feature (F5: Acc); pink, one hydrogen bond donor and acceptor feature (F7: Don&Acc); orange, two aromatic features (F6 and F9: Aro). Peptide 5 is shown in yellow stick form; active site residues (green) are shown in line form; red balls mean water molecules; a molecular surface is colored by H-bonding (orange), hydrophobicity (green) and mild polar (blue) regions of the binding site in PLK1-PBD.

**Figure 4:**
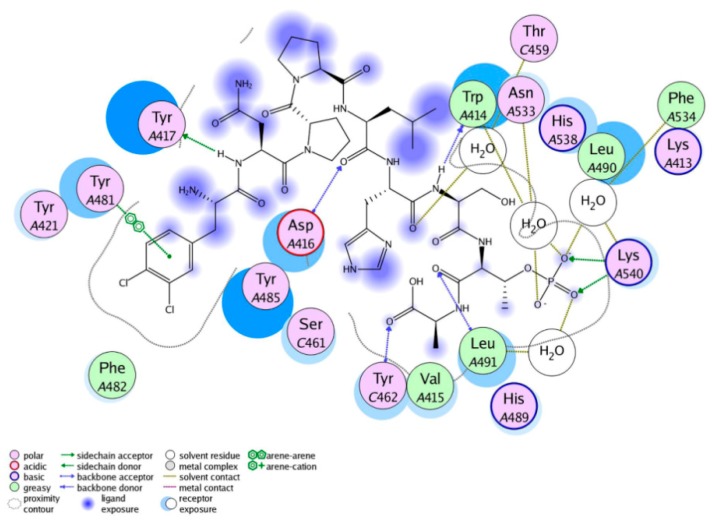
The ligand–protein interaction diagram for the binding site of PLK1-PBD (PDB ID: 3Q1I) with peptide 5. The active site residues are represented as follows: Polar residues in light purple, hydrophobic residues in green, acidic residues with a red contour ring, basic residues with a blue contour ring. Green and blue arrows indicate hydrogen bonding to side chain and backbone atoms respectively. The π–π stacking interactions are represented as green dotted lines. Light-blue “halos” around residues indicate the degree of interaction with ligand atoms (larger and darker halos means more interaction).

In order to predict a reasonable binding mode, the most potent compound, peptide 5 was docked into the active site of PLK1-PBD. It should be noted that the ligand-binding site of PLK1-PBD consists of a hydrophobic pocket and a positively charged binding pocket. The most commonly reported peptide inhibitors including “HSpTA” motif only bound to the positively charged binding pocket of PLK1-PBD [17]. The MOE docking results of peptide 5 suggested that there were two major interactions between peptide 5 and the PLK1-PBD active site (Figure 3 and Figure 4): (i) The C-terminal phosphorylated threonine bound to the positively charged binding pocket formed multiple hydrogen-bonding interactions with Lys540 and water molecules that were indispensable to the ligand binding of the PLK1-PBD [17]; (ii) The N-terminal 3,4-dichlorophenylalanine bound to the hydrophobic pocket of PLK1-PBD was engaged in a strong hydrophobic interaction with some amino acids, including Tyr481, Tyr421, Phe482 and Tyr485, which indicated that it played a key role in stabilizing peptide 5 in the hydrophobic pocket (Figure S2).

### 2.4. Effect of Peptide 5 on Cycle Arrest in HeLa Cells

Owing to the poor cellular penetration of PLK1-PBD inhibitors, many of them are unable to interfere with the function of PLK1 in cancer cells and thus induce cell-cycle arrest. Peptide 5 was next tested for its ability to perturb PLK1-PBD function during cell signaling. We used flow cytometry to assess the effect of peptide 5 on the cell cycle. Various concentrations of peptide 5 (1, 5, and 25 μM) increased the percentage of HeLa cells in the G2/M phase in a dose-dependent manner (Figure S3a,b). The results indicated that peptide 5 induced HeLa cell cycle arrest at the G2/M phase. To investigate the effect of peptide 5 on the function of PLK1-PBD, we monitored the p-Cdc25C levels by immunoblotting with specific antibodies. Peptide 5 significantly decreased the p-Cdc25C protein levels in HeLa cells (Figure S3c,d). Expression levels of the G2/M phase regulatory proteins were assayed to determine the molecular mechanism of the peptide 5-induced G2/M phase arrest in HeLa cells. Compared to the control group, peptide 5 treatment significantly decreased the cyclinB1 and CDK1 protein levels in HeLa cells (Figure S2c,d). These results suggested that peptide 5 could inhibit the phosphorylation of p-Cdc25C and cell cycle regulatory proteins by affecting the PLK1-PBD, thereby inducing mitotic arrest at the G2/M phase.

## 3. Materials and Methods

### 3.1. Pharmacophore Model Generation and Validation

Three X-ray crystallographic structures of the PLK1-PBD domain, with a resolution of less than 2 Å (PDB code: 3Q1I, 3P36 and 3FVH), were obtained from the Protein Data Bank (PDB) database. These protein structures were first prepared by adding the hydrogen atoms using the Prepare Protein tool, and missing amino acids of ligands were added using the builder tool of MOE. After a series of energy minimizations were carried out with the Merck molecular force field 94 (MMFF94), these proteins were used for generating the most representative features of the PLK1-PBD active site using the pharmacophore generation editor protocol within the molecular operating environment (MOE) (Chemical Computing Group Inc., Montreal, QC, Canada). The resulting structure-based pharmacophore model contains the important pharmacophore features, which are indicated as spheres that represent the essential interaction points with the key residues on the inhibitor binding of the PLK1-PBD.

The Gunner–Henry (GH) scoring method was used to assess the quality of the pharmacophore model [19]. A database containing 1985 inactive and 15 active peptides was constructed. The 15 active peptides are collected from the reported literature [20,21] (Table S3). Previous studies have found that a phosphopeptide motif (HSpTA) has a pivotal role in targeting PLK1-PBD [17]. Since the phosphopeptide sequence excluding a “HSpTA” motif is unable to target PLK1-PBD, 1985 peptide sequences without the “HSpTA” were selected as inactive peptides from the antimicrobial peptide database (http://aps.unmc.edu/AP/). Then, the validated pharmacophore model was used as a 3D query to screen the database containing 2000 compounds using the pharmacophore search protocol available in MOE. Finally, many statistical parameters were calculated, including the total hits (*Ht*), % yield of actives, % ratio of actives, enrichment factor (*E*), and the goodness-of-hit score (*GH*). The GH score range from 0, indicating the null model, to 1, which indicates the ideal model.

### 3.2. Virtual Screening

Since a peptide motif (HSpTA) plays a critical role in perturbing PLK1 function [17], the MOE was applied to build a two-dimensional (2D) database containing ~32,000 phosphorylated peptides with the “HSpTA” motif [22]. The energy minimization algorithm of MOE was then used to convert 2D chemical structures of the database to 3D structures. Before virtual screening, some reported peptides in the peptide database were removed in order to identify novel PLK1-PBD peptide inhibitors [22]. Afterward, the established pharmacophore model was used to screen the phosphorylated peptides from the database by pharmacophore search tool of the MOE. Hit compounds (hit list) were ranked according to the root mean square distance (RMSD) values between the query features of the model and their matching ligand annotation points [23], which is the degree of consistency with the pharmacophore model. In the MOE, lower RMSD values indicate a better mapping of query features and the ligand annotation points. The RMSD value of 0 indicates the ideal mapping. To decrease the number of hits, we used 1 Å of the maximum RMSD value to prune the hit list.

### 3.3. Molecular Docking

The crystal structure of PLK1-PBD (PDB ID: 3Q1I) was obtained from the PDB database. Hydrogen atoms were added to the protein and energy minimization was performed using the MMFF94 force field until an RMSD derivative of 0.001 was achieved. The hits retrieved from databases using virtual screening were docked to the active site of PLK1-PBD using the triangle matcher protocol of MOE program. In the docking calculations, a single 3D conformer of MOE was used to generate conformations. The maximum was terminated when the RMSD gradient fell below the value of 0.01 Å. The maximum number of iterations for minimization was set at 500 kcal/mol. Then, the poses generated by the placement protocol were rescored using the dG docking scoring function of MOE [23,24]. Finally, according to the calculated docking scores, the selected peptides were subjected to biological evaluation.

### 3.4. In Vitro PLK1-PBD Inhibition Assay

A total of 20 μL of 200 nM human PLK1-PBD protein in phosphate-buffered saline (PBS, pH 7.4) was mixed with 20 μL of tested peptides at various concentrations in each well of a 384-well black bottom plate (Corning 3575, Thermo Scientific, Massachusetts, USA). Then, 20 μL of 30 nM probe was added to each well. The plate was shaken at room temperature for 40 min. The polarization values in millipolarization units (mP) were measured at excitation/emission wave lengths of 485/535 nm using a SpectraMax Paradigm Multi-Mode Detection Platform (Molecular-Devices, Tokyo, Japan). The percentage inhibition of the tested peptides at each concentration was defined as IC_50_ = 1 − (P_obs_ − P_min_)/(P_max_ − P_min_). Pmax is the polarization of the wells containing PLK1-PBD and the probe, Pmin is the polarization of the free probe, and the Pobs is the polarization for the wells containing the peptides with a series of concentrations. According to a previously reported method [20], FITC-GPMQSpTPLNG-OH was used as the fluorescein-labeled peptide, which was dissolved in dimethyl sulfoxide (DMSO) and the final optimized concentration was set at 20 nM. Phosphopeptides used for competition binding assays were dissolved in assay buffer. The buffer is made up of 50 mM NaCl, 10 mM Tris (pH 8.0), 1 mM EDTA and 0.1% Nonidet P-40. We further performed the sensitivity by synthesizing two fluorescent probes, FITC-GPMQTSpTPKNG-OH for PLK2-PBD and FITC-GPLATSpTPKNG-OH for PLK3-PBD, respectively. All experiments were performed in triplicate and the data were analyzed using GraphPad Prism 6.0 software (GraphPad Software, San Diego, CA, USA).

### 3.5. Cell Cycle Assay and Western Blot Analysis

HeLa cells were seeded in 6-well culture plates with 5 × 10^5^ cells per well and incubated for 24 h. The cells were treated with the control vehicle (0.01% DMSO) of 1 μM, 5 μM and 25 μM peptide 5 for 72 h. The treated cells were harvested, washed with ice-cold PBS, and then fixed with 70% ethanol overnight at 4 °C. Subsequently, the cells were resuspended in 1 mL of PBS containing 1 mg/mL RNase and 50 μg/mL propidium iodide and incubated at 37 °C for 30 min. Cell cycle distribution of nuclear DNA was analyzed by Accuri C6 flow cytometer (BD Biosciences, Franklin Lakes, NJ, USA). After treatment with peptide 5 for 72 h, HeLa cells were washed and lysed with RIPA lysis buffer. Protein samples were detected by western blot, as described previously [20]. Anti-actin antibody was used as loading control antibody for the total protein samples. The relative levels of protein were analyzed using Image J software (National Institutes of Health, Bethesda, MD, USA).

## 4. Conclusions

We aimed to identify potent PLK1-PBD peptide inhibitors with cellular function by structure-based pharmacophore- and docking-based virtual screening. The 9 peptides discovered by virtual screening were identified as the hit inhibitors of PLK1-PBD. Peptide 5 is the most potent inhibitor and tested for its ability to perturb PLK1-PBD function during cell signaling. Unlike most PLK1-PBD peptide inhibitors with poor cellular penetration, peptide 5 is a potential inhibitor that induces mitotic arrest in tumor cells. Together, this work provides clues for the future development of specific peptide inhibitors that perturb PLK1 function during cell signaling.

## Figures and Tables

**Table 1:** Pharmacophore model validation by goodness-of-hit score (GH) score method.

Serial No.	Parameter	Pharmacophore Model
1	Total molecules in database (*D*)	2000
2	Total number of actives in database (*A*)	15
3	Total hits (*Ht*)	18
4	Active hits (*Ha*)	13
5	% Yield of actives [(*Ha*/*Ht*) × 100]	72%
6	% Ratio of actives [(*Ha*/*A*) × 100]	87%
7	Enrichment factor (*E*) [(*Ha* × *D*)/(*Ht* × *A*)]	96
8	False negatives [*A* − *Ha*]	2
9	False positives [*Ht − Ha*]	5
10	Goodness of hit score (*GH*) ^a^	0.76

^a^ (*Ha*(3*A* + *Ht*)/4*HtA*)(1 − (*Ht* − *Ha*)/(*D* − *A*)); *GH* score of 0.7–0.8 indicates a very good model.

**Table 2:** Results of root mean square distance (RMSD) values and docking scores of the 9 selected peptides.

Peptides	Sequence ^a^	RMSD [Å] ^b^	Docking Score [kcal/mol] ^c^
1	YEPPLHSpTAIG	0.26	−24.54
2	WDPPLHSpTAI	0.38	−23.85
3	FEPPLHSpTAI	0.44	−21.94
4	FEPPLHSpTAG	0.23	−25.36
5	ΦNPPLHSpTA	0.36	−23.31
6	WAPPLHSpTAK	0.45	−20.96
7	WKPPLHSpTAG	0.47	−20.87
8	HKPPLHSpTA	0.51	−20.13
9	HQPPLHSpTA	0.53	−20.07

^a^ Φ, *L*-3,4-dichlorophenylalanine; ^b^ notation points (lower RMSD values indicate a better mapping of query features and the ligand annotation points); ^c^ Docking score between PLK1-PBD and a peptide ligand (lower values indicate a better binding affinity).

## Supplementary Materials

The following are available online. Figure S1: Pharmacophore mapping of 9 peptides on the model. Figure S2: Key interactions of the 3,4-dichlorophenylalanine involved in stabilizing peptide 5 in the hydrophobic pocket of PLK1-PBD (PDB ID: 3Q1I). Figure S3: The effect of peptide 5 on cycle arrest in HeLa cells. Table S1: Results of docking scores and IC_50_ values of the 9 selected peptides. Table S2: Selectivity of peptide 5 against PLKs-PBD. Table S3: Sequences of 15 active peptides.
