# Supplementary material for: Structure-Based Virtual Screening and Biological Evaluation of Peptide Inhibitors for Polo-Box Domain
Source: Molecules. 2019 Dec 27;25(1):107. doi: 10.3390/molecules25010107 (PMC6982974; doi:10.3390/molecules25010107)
Supplement: Supplementary file 1 [file molecules-25-00107-s001.pdf]

Supplementary Materials for

Structure-Based Virtual Screening and Biological Evaluation of Peptide Inhibitors for Polo-Box Domain

*Fang Yan, Guangmei Liu, Tingting Chen, Xiaochen Fu and Miao-Miao Niu**

Department of Pharmaceutical Analysis, China Pharmaceutical University, Nanjing, 210009 (China)

** Correspondence: niumm@cpu.edu.cn (M.-M.N.); Tel.: +8625-8327-1080 (M.-M.N.)*

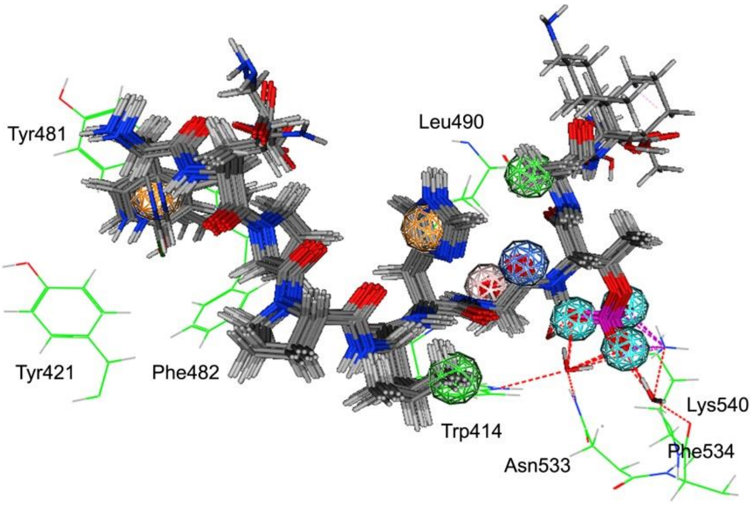

Figure S1. Pharmacophore mapping of 9 peptides on the model. Pharmacophore features are color-coded: Green, two hydrophobic feature (F1 and F8: Hyd); cyan, three anionic and hydrogen bond acceptor features (F2-F4: Ani&Acc); blue, one hydrogen bond acceptor feature (F5: Acc); pink, one hydrogen bond donor and acceptor feature (F7: Don&Acc); orange, two aromatic features (F6 and F9: Aro).

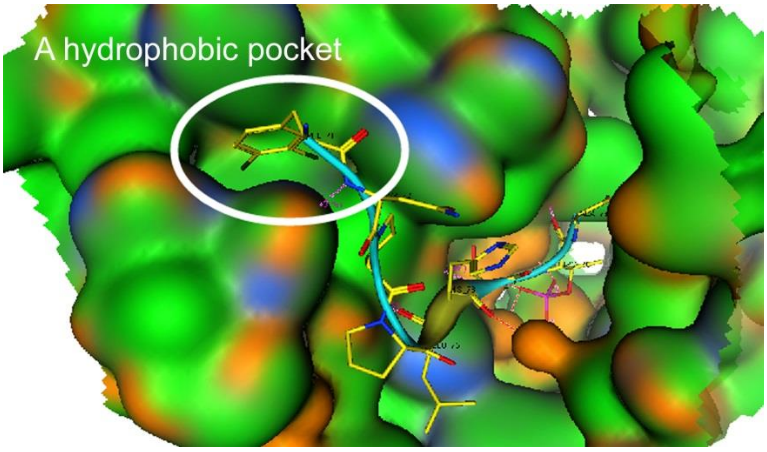

Figure S2. Key interactions of the 3,4-dichlorophenylalanine involved in stabilizing peptide 5 in the hydrophobic pocket of PLK1-PBD (PDB ID: 3Q1I). The peptide 5 is shown in yellow stick form; the hydrophobic pocket of PLK1-PBD is colored by a white line.

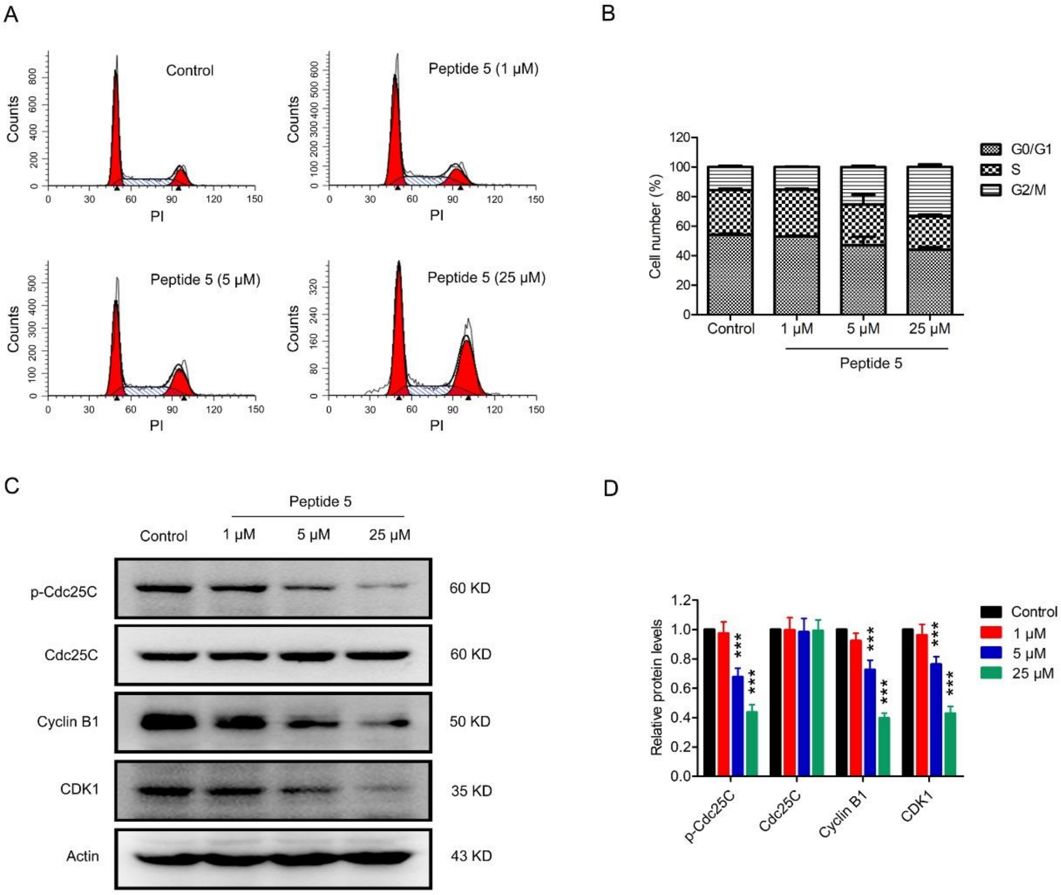

Figure S3. The effect of peptide 5 on cycle arrest in HeLa cells. (A-B) The G2/M arrest induced by peptide 5 in HeLa cells. (C-D) Western blot analysis of p-Cdc25C, Cdc25C, cyclinB1 and CDK1 protein levels in HeLa cells treated with peptide 5. Data reported represent the mean ± SD of three independent experiments. ***$P < 0.001$.

Table S1. Results of docking scores and IC_{50} values of the 9 selected peptides.

Peptides	Sequence [a]	Docking sore [kcal/mol] [b]	IC_{50} [μM]
1	YEPPLHSpTAIG	-24.54	0.32 ± 0.02
2	WDPPLHSpTAI	-23.85	0.19 ± 0.01
3	FEPPLHSpTAI	-21.94	0.29 ± 0.02
4	FEPPLHSpTAG	-25.36	0.16 ± 0.02
5	ΦNPPLHSpTA	-23.31	0.07 ± 0.006
6	WAPPLHSpTAK	-20.96	0.35 ± 0.11
7	WKPPLHSpTAG	-20.87	0.42 ± 0.20
8	HKPPLHSpTA	-20.13	0.61 ± 0.32
9	HQPPLHSpTA	-20.07	0.72 ± 0.24
Poloboxtide	MAGPMQSpTPLNGAKK	-19.84	7.92 ± 1.03

[a] Φ, *L*-3,4-dichlorophenylalanine; [b] Binding free energy between PLK1-PBD and a peptide ligand (Lower values indicate a better binding affinity).

Table S2. Selectivity of peptide-5 against PLKs-PBD.

Peptides	PLK1-PBD	PLK2-PBD	PLK3-PBD
Peptide 5	70 nM	NA[a)]	NA

[a)]NA, no significant inhibition at 1 μM inhibitor. All measurements were performed in triplicates and the IC_{50} values represent the mean ± SD of three data sets.

Table S3. Sequences of 15 active peptides.

Peptides	Sequence [a]
1	Ac-QTF(4-NO2)DPPLHSpTAIYAN-NH2
2	Ac-QTF(4-OCH3)DPPLHSpTAIYAN-NH2
3	Ac-QTF(3,4-Cl)DPPLHSpTAIYAN-NH2
4	Ac-TF(3,4-Cl)DPPLHSpTAIYAN-NH2
5	Ac-QF(3,4-Cl)DPPLHSpTAIYAN-NH2
6	Ac-F(3,4-Cl)DPPLHSpTAIYAN-NH2
7	Ac-QTF(4-Cl)DPPLHSpTAIYAN-NH_2
8	Ac-FDPPLHSpTAIYAN-NH2
9	Ac-QTF(4-F)DPPLHSpTAIYAN-NH2
10	FDPPLHSpTA-NH_2
11	WDPPLHSpTA-NH_2
12	LDPPLHSpTA-NH_2
13	VDPPLHSpTA-NH_2
14	ZDPPLHSpTA-NH_2
15	HDPPLHSpTA-NH_2

[a] Z, 3-(3,4-dichlorophenyl) propionic acid.
